# Body and Hand–Object ROI-Based Behavior Recognition Using Deep Learning

**DOI:** 10.3390/s21051838

**Published:** 2021-03-06

**Authors:** Yeong-Hyeon Byeon, Dohyung Kim, Jaeyeon Lee, Keun-Chang Kwak

**Affiliations:** 1 Interdisciplinary Program in IT-Bio Convergence System, Department of Electronics Engineering, Chosun University, Gwangju 61452, Korea; qasdfghjt@hanmail.net; 2 Intelligent Robotics Research Division, Electronics Telecommunications Research Institute, Daejeon 34129, Korea; dhkim008@etri.re.kr (D.K.); leejy@etri.re.kr (J.L.)

**Keywords:** behavior recognition, convolutional neural network, skeleton, RGB video, ensemble

## Abstract

Behavior recognition has applications in automatic crime monitoring, automatic sports video analysis, and context awareness of so-called silver robots. In this study, we employ deep learning to recognize behavior based on body and hand–object interaction regions of interest (ROIs). We propose an ROI-based four-stream ensemble convolutional neural network (CNN). Behavior recognition data are mainly composed of images and skeletons. The first stream uses a pre-trained 2D-CNN by converting the 3D skeleton sequence into pose evolution images (PEIs). The second stream inputs the RGB video into the 3D-CNN to extract temporal and spatial features. The most important information in behavior recognition is identification of the person performing the action. Therefore, if the neural network is trained by removing ambient noise and placing the ROI on the person, feature analysis can be performed by focusing on the behavior itself rather than learning the entire region. Therefore, the third stream inputs the RGB video limited to the body-ROI into the 3D-CNN. The fourth stream inputs the RGB video limited to ROIs of hand–object interactions into the 3D-CNN. Finally, because better performance is expected by combining the information of the models trained with attention to these ROIs, better recognition will be possible through late fusion of the four stream scores. The Electronics and Telecommunications Research Institute (ETRI)-Activity3D dataset was used for the experiments. This dataset contains color images, images of skeletons, and depth images of 55 daily behaviors of 50 elderly and 50 young individuals. The experimental results showed that the proposed model improved recognition by at least 4.27% and up to 20.97% compared to other behavior recognition methods.

## 1. Introduction

In modern society, it is possible to preserve health by restoring age-deteriorated bodily functions to a certain level through technologies including medicine and engineering. Advances in these technologies have led to an increase in life expectancy and subsequently a rise in the elderly population. Furthermore, the elderly population ratio is rapidly increasing, due to the lower number of newborns resulting from the decline in the birth rate, and because, over time, young people continue to move into the elderly generation group. The burden on the earning population and the government is expected to increase if the number of young individuals is significantly reduced compared to the number of elderly individuals. The accompanying structural change renders an increasing elderly population a problem in modern society [1,2,3].

In the past, most people had occupations in which physical labor was important, such as agriculture, commerce, and fishing. Most of the time was spent in securing food, clothing, and shelter. Extended families became a social unit due to these economic activities. The extended family system is characterized by the sharing among various family members of the care of elderly people, whose health deteriorates and who become incapable of economic activity as they age. However, in modern society, the dependence on the extended family system has declined compared to the past due to the abundance of food, clothing, and shelter, and the diversification of occupational groups. Care for the elderly is changing into a social role as family members are scattered due to the rise of nuclear families [4,5]. Because caring for the elderly is repetitive labor and difficult depending on the situation, society and the government have been working on research and development for home service “silver robots” to replace humans in this work. Because the environment these robots face is complex, unlike the simple movement of factory manufacturing robots, silver robots require advanced artificial intelligence technology to respond appropriately to the aged [6,7,8,9,10].

Behavior recognition technology automatically recognizes the behavior of an actor by analyzing input data from cameras and inertial sensors. The environment that a person is in can be understood and responded to appropriately by recognizing the behavior of the person through behavior recognition. For example, elderly care can be automated if the home service silver robot is able to determine or recognize sudden fainting and other ill health symptoms of the elderly, and perform appropriate actions while attending the elderly, who are alone [11,12]. 

Due to the recent progress of deep learning, a framework has been established for computers to automatically process existing complex problems. Deep learning involves building hidden layers deeply in existing neural networks and training them using a backpropagation algorithm, which efficiently solves nonlinear problems. Studies have applied such deep learning technology to behavior recognition [13,14]. However, the results of down sampling of an image are poor, because detailed information from an image is lost, and the inference time increases if a high-resolution image is used without modification. Therefore, Karpathy [15] proposed the fusion of two streams running in parallel for video classification. Two encoders running in parallel were made smaller to simplify the parameters. One encoder was a low-resolution encoder, and the other processed high-resolution images; the results of both were merged in the last fully connected layer. Although the fusion approach works well for short videos, it is challenging to classify long videos, as many frames must be computed and many aspects must be memorized. Ng [16] proposed two methods to classify long videos. The first uses maxpooling for the time axis of the convolutional features, and the second concatenates the convolutional features with long short-term memory (LSTM) to process videos of various lengths. In a video, the motion of an object yields good information about the action it performs; this motion can be measured using optical flow. Simonyan [17] proposed a behavior recognition method using two streams from the image and optical flow. One stream inputs individual frames, whereas the other calculates the optical flow using several frames. Subsequently, the scores of the two are combined at the end by inputting each of them to a convolutional neural network (CNN). The two-dimensional (2D) convolution takes 2D data and outputs a 2D result, whereas three-dimensional (3D) convolution can output a 3D result by inputting 3D data because it performs a convolution operation in three directions. Tran [18] proposed a 3D convolutional neural network structure based on 3D convolutional operation for video behavior recognition. The network has eight convolution layers and two fully connected layers. Wang [19] used the trajectory of the body part to classify the actions performed. In that study, the handcrafted features of Fisher vectors and the deep-learned features based on CNN were combined in the last layer after the trajectory was extracted from the video. Yang [20] proposed a multimodal combination with four models for video classification. These four models are the 3D convolution feature, 2D optical flow, 3D optical flow, and 2D convolution feature. A boosting mechanism was used for the fusion method. Another scheme used for behavior recognition, the attention mechanism, gives more weight to a specific area than other areas as a method of paying attention to the area for recognition activity. These weights are learned from data and are generally divided into soft and hard methods. The soft is a decisive method, and the hard is probabilistic. Shama [21] applied an attention mechanism for video classification. The position probability was obtained by inputting the convolution feature map and the position weight serially to three LSTMs. This attention not only improved accuracy, but also provided a way to visualize predictions. 

Several studies have been conducted on skeleton-based behavior recognition. Recurrent neural networks (RNNs) exhibit gradient vanishing and exploding problems; moreover, they have difficulties in learning and long-term patterns. To this end, LSTM and gated recurrent units (GRU) were developed; however, the use of hyperbolic tangent and sigmoid functions resulted in gradient decay over layers. Therefore, Li [22] proposed indRNN, wherein neurons in the same layer are independent of each other and are connected across layers. This network can be stacked deeper than conventional RNNs and can process longer sequences. Skeleton-based behavior recognition was performed on this network using the Nanyang Technological University (NTU) RGB+D (red-green-blue +depth) dataset. However, skeleton-based behavior recognition has limitations in large datasets due to its limited ability to represent features; recent RNNs have been developed with a focus on changes in body joints over time, without considering geometric relationships. Wang [23] introduced joints, edges, and surfaces to reflect the geometric relationship between joints for behavior recognition. These three geometric aspects were used as inputs to a general RNN, using a viewpoint transformation layer and a temporal dropout layer. Moreover, the multi-scale sliding window algorithm was used by classifying the behavior by frame for behavior detection. Most recent behavior recognition methods using skeletons are based on RNN. Li [24] proposed a novel CNN for behavior recognition and behavior detection. Raw skeleton coordinates and skeleton motion are fed into the CNN. A new skeleton transformer module was designed to rearrange and select important skeleton joints automatically. A window proposal network that extracts temporal segment proposals for behavior detection was developed. The dynamics of the human body skeleton convey significant information for behavior recognition. Conventional approaches for modeling skeletons relied on handcrafted parts, thus resulting in limited ability to represent the skeleton and difficulties of generalization. Therefore, Yan [25] extracted the skeleton information for each frame from the image and presented the information in a skeleton graph with a temporal dimension, thereby classifying it using a spatio-temporal graph convolutional network (ST-GCN).

Several studies have been conducted on attention-based behavior recognition. It is important to extract discriminative spatio-temporal features to model the evolutions of different behaviors. Song [26] proposed a spatio-temporal attention model to explore and detect discriminative spatio-temporal features for behavior recognition from skeletons. An RNN-based model was designed with LSTM units. The trained model was capable of selectively focusing on the discriminative joints of the skeleton in each input frame and paying different levels of attention to the output of different frames. For efficient training, a regularized cross-entropy loss and joint training strategy were proposed. Further, a method of generating behavior temporal proposals for behavior detection was developed based on temporal attention. Behavior recognition with a three-dimensional skeleton sequence has gained a reputation for speed and robustness. The recently proposed CNN-based method also showed good performance in learning spatio-temporal features. Nevertheless, there are two problems that potentially limit performance. First, previous skeleton representations are generated by chaining joints in a fixed order. The corresponding semantic meaning is unclear, and structural information is lost among the joints. Second, previous models do not have the ability to focus on informative joints. The attention mechanism is important in skeleton-based behavior recognition because other joints contribute non-uniformly to accurate recognition. Yang [27] redesigned the skeleton representation with a depth-first tree order to enhance the semantic meaning of the skeleton image and better preserve the associated structural information. Further, a general two-branch attention architecture was proposed that automatically focuses on spatio-temporal key stages and filters out unreliable joint prediction. Based on the proposed general structure, a global long sequence attention network with an improved branch structure was designed. A sub-sequence attention network (SSAN) was proposed that takes a sub-image sequence as an input to adjust the kernel’s spatio-temporal aspect ratio and better extract long-term dependence. The two-branch attention structure was further improved by combining it with SSAN. 

Behavior recognition research using object information has also been conducted. Moore [28] introduced a framework for recognizing behavior and objects by measuring image-based, object-based, and behavior-based information from videos. Hidden Markov models were combined with object context to classify hand actions. Furthermore, the Bayesian method was used to differentiate the class of unknown objects by evaluating detected behaviors along with low-level, extracted object features. Most of the proposed methods recognize behaviors and objects separately. However, it is important to recognize that behaviors and objects are complementary to each other, as behaviors of, for example, a hand, are related to the objects they grasp. Saitou [29] represented the relationship between behavior and object in a hierarchical model and tracked the movement of the head and hand through vision. The features of behaviors, such as location and direction, were extracted and input to the dynamic Bayesian network to classify behaviors approximately. Then, the behaviors and related objects were refined using a conceptual model. Gu [30] proposed a hierarchical probability model-based framework, which not only models the dynamics of the behaviors, but also the contextual constraints in terms of object/behavior correlation and behavior sequential constraints to improve behavior recognition performance. By considering the behavior/object correlation, even behaviors that are difficult to detect or recognize can be recognized using motion features only. By contrast, the behavior sequential constraints can further improve the recognition accuracy. In the proposed method, first, the dynamics of a behavior was modeled using the hidden Markov model; a Bayesian network was adopted to model the object constraints for low-level behavior recognition. Consequently, a high-level HMM (hidden Markov model) was created to model the sequential constraints, which refine the decision from the Bayesian model. 

Ensemble-related studies with various inputs were conducted. It is difficult to classify behaviors related to objects with similar motions for skeleton-based behavior recognition from depth cameras. Other available video streams (RGB, infrared, depth) provide additional clues. Boissiere [31] proposed a modular network combining skeleton and infrared data. The pre-trained 2D CNN was used as a pose module to extract features from the skeleton data. The pre-trained 3D CNN was used as an infrared module to extract visual features from videos. Both feature vectors were concatenated using a multilayer perceptron. The two-dimensional skeleton coordinates were used to crop the region of interest (ROI) around the subject in the infrared video. Infrared video is less sensitive to illumination and more usable in the dark. Liu [32] considered behavior recognition based on multimodal fusion between 3D skeleton and RGB images. A neural network was designed that uses a 3D skeleton sequence and a single middle frame as input. The self-attention module and skeleton attention module were used. Further, temporal features were extracted from the skeleton sequence via a Bi-directional long short term memory (Bi-LSTM). Moreover, the spatial and temporal features were combined via a feature fusion network. 

Although most of the information for behavior recognition is concentrated in the human domain, redundant information is obtained because multiple images overlap in the RGB video. To solve this problem and increase the recognition rate, a model that diversifies features and finally ensembles the results was proposed by designing a model that focuses on the human part that is important for behavior, and the hand–object interaction, which represents the main information of the behavior. The first stream uses the pre-trained 2D-CNN by converting the 3D skeleton sequence into pose evolution images (PEIs), and the second stream uses the RGB video input to the 3D-CNN to extract temporal and spatial features from RGB. The important information in behavior recognition is the person performing the action. The features can be analyzed by focusing on the action itself rather than when trained with the entire region if the neural network is trained after removing the surrounding noise and placing the ROI on the person. Therefore, in the third stream, the RGB video is limited to the body ROI and input to the 3D-CNN for use. Because humans use tools to perform actions, unlike animals, training a neural network by placing an ROI on the hand–object interaction enables feature analysis by focusing on tool information. Therefore, in the fourth stream, the RGB video is limited to hand–object interaction ROI and input to the 3D-CNN for use. Finally, because better performance can be expected by combining the information of the models trained by focusing on these regions of interest, better recognition can be performed through late fusion of the four stream scores. 

The Electronics and Telecommunications Research Institute (ETRI)-Activity3D database, which has color images, images of skeletons, and depth images of 55 daily behaviors of 50 elderly and 50 young people, was used as the database for the experiment. This dataset is the second largest behavior recognition database, consisting of a total of 112,620 samples. The data were acquired using up to eight multi-directional Kinect v2s in an actual residential environment. Further, the sensors were 70 and 120 cm in height and acquired data at a distance within 1.5 to 3.5 m, assumed to replicate the environment of the silver robot. 

As an experimental result of the proposed method, the accuracy of the 3D-CNN of body ROI input and the 3D-CNN of hand–object interaction ROI input was 76.85% and 73.11%, respectively. The accuracy of the proposed ROI ensemble (Type6) in which 3D-CNN, BodyROI-3D-CNN, and HandObject-3D-CNN (single models of RGB video input), and PEI-T3-2D-CNN (single model of skeleton input), are ensembled, was 94.87%, thereby showing that the accuracy was improved by a minimum of 8.78% and a maximum of 21.76% compared to the single model. Further, the accuracy of the proposed ROI ensemble (Type6) was improved by a minimum of 4.27% and a maximum of 20.97% compared with the methods of other studies. The contributions of this paper are the diversification of features and the improvement of accuracy through an ensemble by paying more attention to the key information of behavior recognition after removing unnecessary information and applying the ROI to the hand–object interaction.

The contribution of this study can be described as follows. First, by focusing on the hand–object and the human body from the skeleton information, the proposed method showed superior recognition performance in comparison to the previous works. Next, we built the ETRI-Activity3D database consisting of a total of 112,620 video samples for behavior recognition. This database is composed of 55 daily behaviors of only elderly and young people in a home environment. Finally, the proposed method can be applied to human–robot interaction in home service robot and silver robot environments.

This study conducted body and hand–object interaction ROI-based behavior recognition using deep learning. The conventional technologies used as sub-technologies of the proposed model are introduced in Section 2. The proposed behavior recognition method is described in Section 3. The experiments conducted to evaluate the performance of the proposed model and their results are described in Section 4, followed by the conclusion in Section 5.

## 2. Techniques for Behavior Recognition

Behavior recognition research has been conducted not only from the perspective of developing artificial intelligence, but also on ways to convert data into behavior recognition efficiently. In this section, the conventional technologies, used as sub-technologies of the proposed model, are introduced. The PEI (pose evolution image) represents a method of converting the coordinate data of a skeleton into image data, and 3D-CNN is a method capable of simultaneously analyzing spatial and temporal features using a three-dimensional filter.

### 2.1. Pose Evolution Image (PEI)

**Figure 1:**
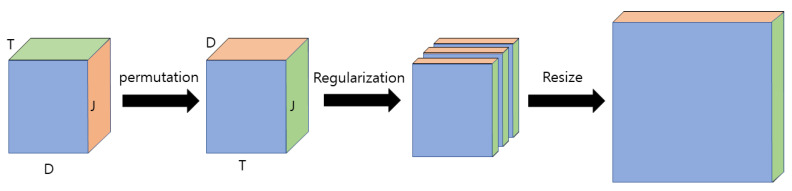
Schematic diagram of imaging process of a skeleton sequence.

**Figure 2:**
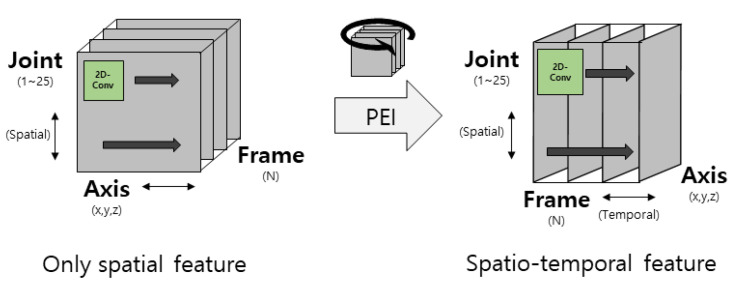
Comparison of feature extraction before and after pose evolution image (PEI) conversion.

The skeleton is a data format that efficiently stores the movements of a person of interest; it is a reconstruction of the human body skeleton into coordinate points based on sensor data. A chronological sequence of skeletons of several moments is created in the form of a video and used for behavior recognition because a skeleton of a moment in image format cannot contain all the behavior information of a person. Transformation methods have been studied to extract the appropriate information effectively, because not only spatial information, but also temporal information, is important to analyze these sequence data effectively. PEI represents a method that converts a skeleton sequence into a single color image. First, because a typical person has limited joints with a central axis at which the body can be folded, the human skeleton can be represented with few data. Kinect v1 represents the human skeleton with 20 joints, whereas Kinect v2 represents the human skeleton with 25 joints. Although detailed changes in the skeleton of a person can be detected when the skeleton is represented by many joints, a skeleton may be incorrectly detected when there are unnecessarily many joints because the human body limitations cannot be considered. A skeleton is a group of these joints, and a 3D skeleton represents the joints of a human body skeleton in 3D coordinates. The skeleton must be detected at every moment as the behavior changes according to the human skeleton as a person moves over time. The resulting skeleton sequence generated for a behavior has a 3D data format. These 3D data are converted to a 2D image by directly projecting the 3D coordinates into RGB space. A schematic diagram of the imaging process of a skeleton sequence is shown in Figure 1. When the skeleton sequence is expressed in (J × D × T) as 3D data, J denotes the number of joints representing the human skeleton; D denotes the number of dimensions of the coordinates representing the joint; and T denotes the number of skeleton frames over time in the temporal dimension. The dimension (D) of the joint coordinates is permutated with the temporal dimension (T) to convert the skeleton sequence into an image. If the number of dimensions (D) of the joint coordinates is three, it results in a single color image (J × T × 3) after the permutation process. A skeleton image is created by regularizing this color image for each channel and linearly converting the image size. Because the pre-trained 2D-CNN is designed to be input with three channels of RGB mainly for image recognition, it can be used directly in the pre-trained 2D-CNN by converting the skeleton sequence to PEI. Further, all of the spatio-temporal features can be considered with only a 2D filter by converting the skeleton sequence to PEI. The feature extraction before and after PEI is compared in Figure 2, and the regularization equation for each channel is as shown in Equation (1). This imaging method is defined as Type 1 [33].

(1) $$x\prime =\frac{x-\min\left(x\right)}{\max\left(x\right)-\min\left(x\right)}$$

**Figure 3:**
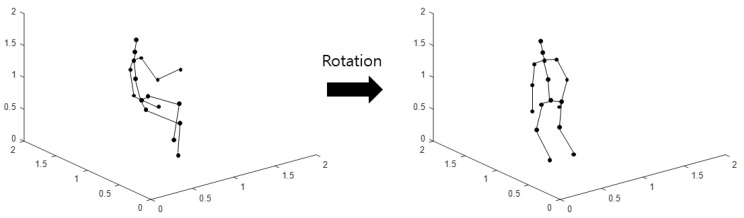
Schematic diagram of rotation of the skeleton.

The aforementioned method of imaging the skeleton sequence can also obtain various images by changing the skeleton data. The rotated coordinates of the rotated skeleton are obtained by rotating the original 3D skeleton coordinates based on the pelvic line; the coordinates are used to perform imaging with the aforementioned method. This imaging method is defined as Type 2. A schematic diagram of the rotation of the skeleton is shown in Figure 3.

**Figure 4:**
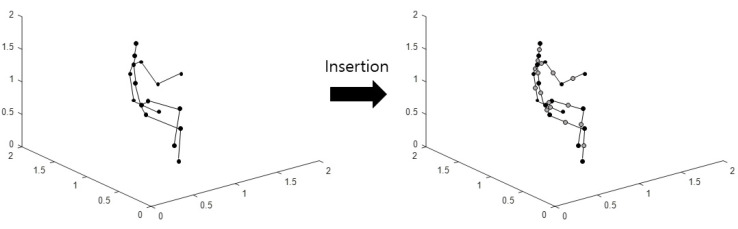
Schematic diagram of skeleton joint insertion.

Imaging is performed using the aforementioned method by inserting new joints between two neighboring coordinates from the original 3D skeleton coordinates. This imaging method is defined as Type 3. A schematic diagram of the joint insertion in a skeleton is as shown in Figure 4.

Finally, imaging is performed using the aforementioned method by applying both rotation and insertion from the original skeleton coordinates in 3D, which is defined as Type 4 [33].

### 2.2. 3D Convolutional Neural Network

In machine learning, a neural network is a method of recognizing digital data by mimicking the structure and operation of the human brain, where logic is created by computers instead of being directly designed by humans. Neurons are nerve cells, which are the structural and functional units of the nervous system; they produce electrical signals and transmit these signals from one part of the body to another as the basic unit of information transmission. Neurons are composed of dendrites, cell bodies, and axons. The dendrites receive external signals and transmit signals to the next neuron through the cell body and axons. In this process, the input signal is transmitted to the next neuron only when the threshold value is exceeded. Neural networks model these functions of the neurons on a computer and use them in artificial intelligence technology. In a neural network, a neuron is defined as a node, and a threshold value is defined as an activation function. Among the numerous neurons, some are strongly tied to each other and some are not tied; this is defined as the weight between nodes. Early neural networks had a shallow structure of layers composed of only nodes. Although this simple structure works for simple problems, learning cannot be performed when the problem is complex. Now, neural network models that can learn complex problems have been developed by deeply stacking layers and adding layers of various functions. There are several basic neural networks depending on the characteristics of the neural network. The CNN [34] is advantageous for image analysis, the RNN is advantageous for sequence data analysis [35], and the graph neural network (GNN) is advantageous for hierarchical data analysis [36]. 

The conventional image processing method implements a signal processing method for feature extraction based on expert knowledge and classifies the extracted features with a classifier, whereas CNN is an algorithm that extracts and classifies features from data. It consists of a convolutional layer applied with a convolutional filter that passes through a 2D space, a subsampling layer that is stable against changes in movement and size, and a fully connected layer and a SoftMax layer for classification to extract features of an image effectively. 

A 2D CNN can only extract spatial features of an image; however, a 3D CNN effectively extracts not only temporal, but also spatial features. Unlike a single image that only has 2D spatial information, it is difficult to extract sufficient features from a video only with 2D convolution because a video has spatial and temporal information as 3D data, where several images are overlapped. The 3D convolution is efficient for 3D data such as video; it can extract both spatial and temporal features because the filter is 3D. In a 3D CNN, the convolution and pooling operations are performed with a 3D filter; the general structure is however the same as that of a 2D CNN. A 3D convolutional equation is shown in Equation (2).

(2) $$\left(f*g\right)\left(i,j,k\right)=\overset{h-1}{\underset{x=0}{\sum }}\overset{w-1}{\underset{y=0}{\sum }}\overset{t-1}{\underset{z=0}{\sum }}f\left(x,y,z\right)g\left(i-x,j-y,k-z\right)$$

**Figure 5:**
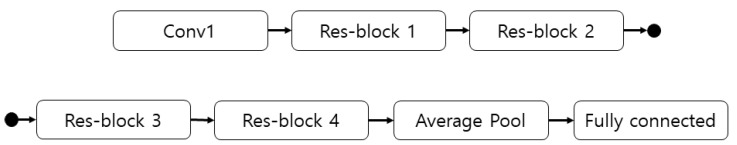
Structure of R3D-18.

Pre-trained models include C3D [18], GoogLeNet-based I3D [37], and ResNet-based R3D [38]. ResNet applies a skip connection that reuses the input features of the previous layer to solve the problems of significant decrease or increase in the slope as the layer deepens, and the resulting degradation of performance. It creates five blocks, where one is used as an input, and the others are stacked in order [39]. Figure 5 shows the schematic structure of R3D-18.

## 3. Proposed Behavior Recognition Method

Behavior recognition data may consist only of RGB video, but generally they also include skeleton sequences in many cases. RGB video is data obtained by photographing several consecutive RGB images at regular intervals. A video is a series of consecutively-photographed images, shown at speeds to make it appear as if the photographed scenario is observed in real time. Skeletons represent human skeleton information extracted from sensor data; they are composed of joint coordinates such as head, shoulders, hands, and feet, and are defined at every frame to form a skeleton sequence. Although the RGB video has a significant difference in data size depending on the resolution of an image, and generally has a file size several tens of times larger than other data, it contains various information including surrounding objects and contexts. By contrast, the skeleton data are small and have only human skeleton structure information because the skeleton data only has joint coordinate information. Although the important information for behavior recognition is the movement in the human skeleton, there are cases where it is necessary to determine the behavior based on the surrounding situations because skeletal information alone is insufficient in the case of similar behaviors. Because the characteristics of these two types of data are different, a better synergy effect is created through adequate assembling of these two data. 

The RGB video has a 3D structure; as 2D images are stacked along the time axis, a 3D-CNN rather than a 2D-CNN must be used. Because the 3D CNN has a 3D filter, all spatio-temporal information is considered, even if it does not correspond to the sequence. The convolution operation and subsampling have a 3D filter; other configurations are the same as in a 2D-CNN. A pre-trained model can achieve good performance by being designed in the same way as a 2D-CNN. 

**Figure 6:**
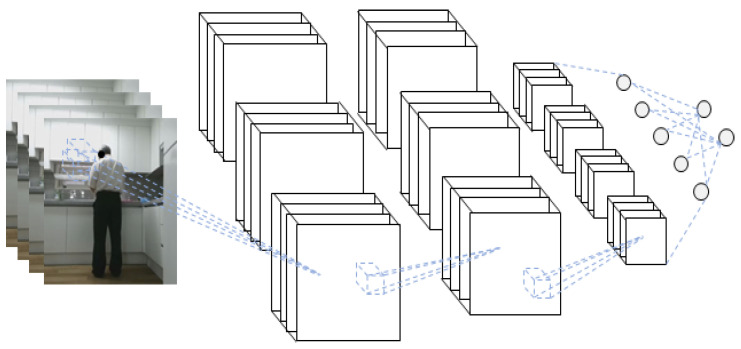
Schematic diagram of the three-dimensional convolutional neural network (3D-CNN) for RGB video input.

The schematic diagram of the 3D-CNN of the RGB video input is shown in Figure 6.

**Figure 7:**
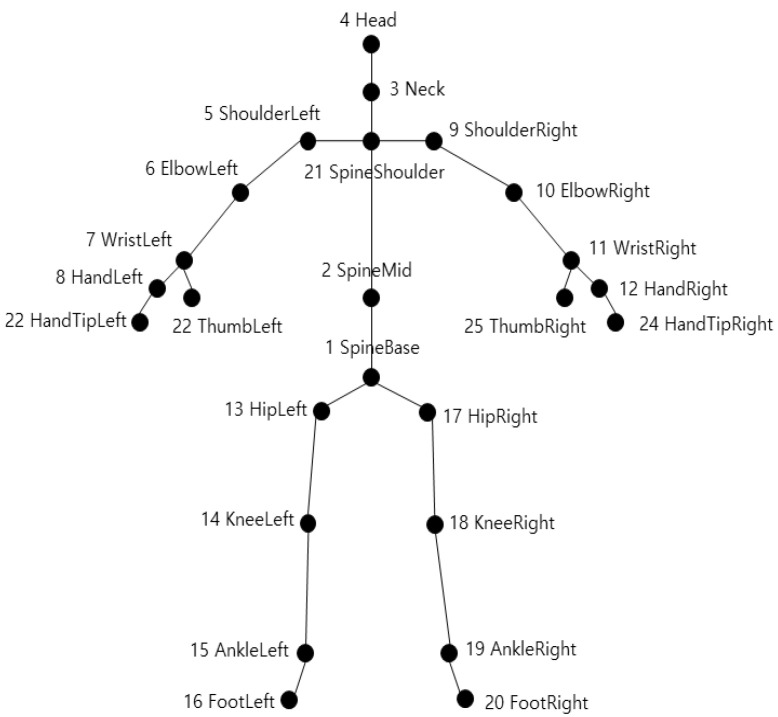
Skeleton joint position of Kinect v2.

Because human joints move in a rotational axis, humans can be modeled with skeleton data if the joints are well designed. For example, Kinect v2, which is widely used to acquire skeleton data, models a human with 25 joints. Kinect v2 acquires joint points with 3D coordinates; the defined joints are shown in Figure 7 [40].

**Figure 8:**
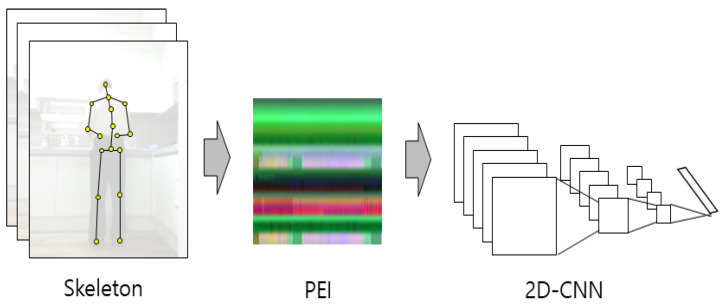
Schematic diagram of the 2D-CNN of the PEI input.

These skeleton data also have a 3D data format with the addition of the time axis when 25 joints are defined by the 3D coordinates of the skeleton data. The joints are converted into a 2D image when these 3D skeleton data are converted using the PEI method. This 2D image can be classified by training a 2D-CNN. A 2D-CNN can use a pre-trained model, such as GoogLeNet, as a feature extractor and classifier. A schematic diagram of the 2D-CNN of the PEI input is shown in Figure 8. As mentioned earlier, four types of PEI are generated by changing the original skeleton data, and four 2D-CNN models can be obtained by training models for each type.

Humans view a scene and recognize objects through the light entering their eyes. They do not observe everything in the scene simultaneously, and recognize objects by focusing on each part of interest. This reduces mistakes and increases accuracy by ignoring unnecessary information and focusing more on the target of interest for the target process. In the RGB video data for behavior recognition, there may not only be the landscape of the place where the action is performed by a person, and the tools used, but also numerous objects. Because the person who performs the action has the key information rather than the surrounding landscape and surrounding objects, better behavior recognition performance is achieved if unnecessary information is removed, and analysis is performed by focusing only on the human body part. Setting the human body part as an ROI is referred to as the body ROI in this study. Further, the human hand area provides important information for behavior recognition because people use tools to perform actions. Setting the hand part as the ROI is referred to as the hand–object ROI in this paper. 

The position of interest must be specified by recognizing a person in the RGB image to extract the body ROI from an RGB video. The joint coordinates are obtained using OpenPose, which extracts skeleton information from deep learning-based RGB images [41]. OpenPose is open software that recognizes human skeletons from RGB images and returns 2D joint coordinates. 

**Figure 9:**
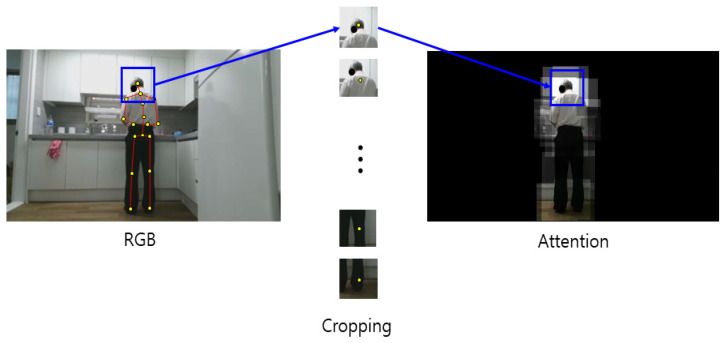
Body region of interest (ROI) extraction process of RGB video using the skeleton.

**Figure 10:**
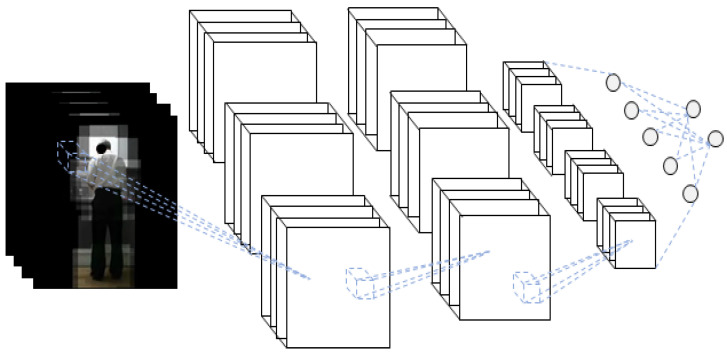
Schematic diagram of the 3D-CNN of the RGB video input with the body ROI.

**Figure 11:**
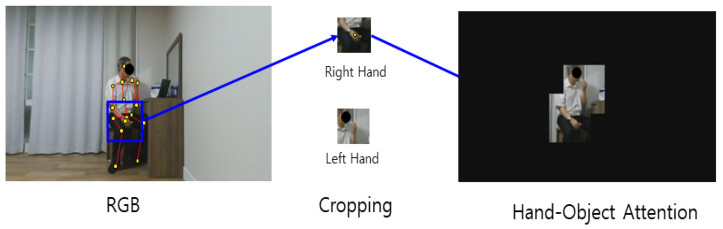
Hand–object ROI extraction process of RGB video using the skeleton.

**Figure 12:**
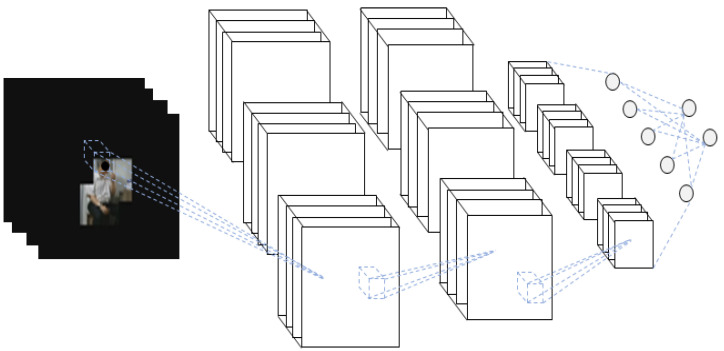
Schematic diagram of the 3D-CNN of RGB video input with the hand–object ROI.

To designate only the body ROI in the RGB video, only the human part is left using the skeleton data and setting other pixel values to zero. To leave only the body part, a certain size box section on the left and right centered on the joint coordinates are copied and pasted in the same coordinates on the blank image of the same size. Only the human body part is copied to the blank image, as this process is performed for all joints. Body ROI data are prepared by removing the background for every frame and converting the data into video again. The hand–object ROI data are prepared by performing the aforementioned process only for the human hand. The process of extracting the body ROI of RGB video using a skeleton is shown in Figure 9. The data prepared in this way are an RGB video in which the background of the ROI is removed, which represents 3D data where images are stacked along the time axis. The 3D-CNN is used as a method of classification. A schematic diagram of the 3D-CNN of the RGB video input with the body ROI is shown in Figure 10. Likewise, the process of extracting the hand–object ROI from an RGB video using the skeleton is shown in Figure 11. The data prepared in this way are 3D data from an RGB video in which the background of the ROI is removed, where images are stacked along the time axis. The 3D-CNN was used as a method of classification. The schematic diagram of the 3D-CNN of the RGB video input with the hand–object ROI is shown in Figure 12.

**Figure 13:**
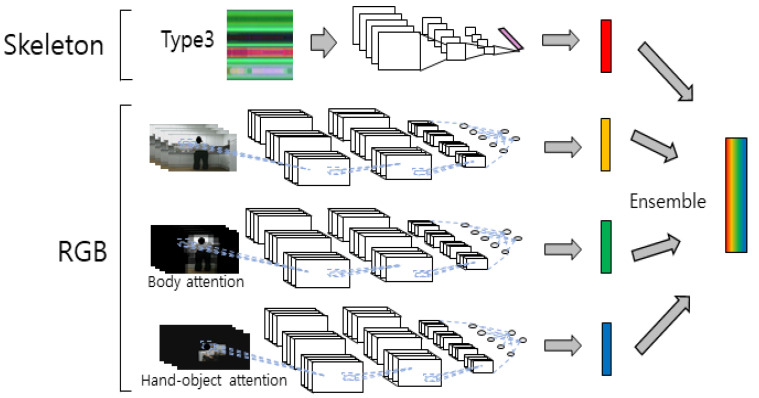
Diagram of ROI-based four-stream ensemble model for behavior recognition.

An ensemble of neural networks is a method to derive better results by combining the results of several individually trained models with one goal. Individual models focus on their respective features without distraction from different input data. Further, individual models can diversify data analysis strategies through different neural network structures. A better synergy effect is created through ensembled models of these various inputs and analysis strategies. A diagram of the ROI-based four-stream ensemble model for behavior recognition is shown in Figure 13. We used the commonly known addition and multiplication for the scores obtained from each stream in the ensemble deep learning model. The addition and multiplication of the output score are defined by Equations (3) and (4), respectively.

(3) $${\mathrm{Output}}_{addition}=max\left(\left[\begin{array}{c} \begin{array}{c} p_{11}\\ p_{12}\\ \vdots \end{array}\\ p_{1m} \end{array}\right]+\left[\begin{array}{c} \begin{array}{c} p_{21}\\ p_{22}\\ \vdots \end{array}\\ p_{2m} \end{array}\right]+\cdots +\left[\begin{array}{c} \begin{array}{c} p_{l1}\\ p_{l2}\\ \vdots \end{array}\\ p_{lm} \end{array}\right]\right)$$

(4) $${\mathrm{Output}}_{multiplication}=max\left(\left[\begin{array}{c} \begin{array}{c} p_{11}\\ p_{12}\\ \vdots \end{array}\\ p_{1m} \end{array}\right]\;\times \;\left[\begin{array}{c} \begin{array}{c} p_{21}\\ p_{22}\\ \vdots \end{array}\\ p_{2m} \end{array}\right]\;\times \;\cdots \;\times \;\left[\begin{array}{c} \begin{array}{c} p_{l1}\\ p_{l2}\\ \vdots \end{array}\\ p_{lm} \end{array}\right]\right)$$

where l and *m* indicate the number of classification models and the number of classes, respectively.

## 4. Experimental Result

This section describes the details of the ETRI-Activity3D dataset used for the experiment, the evaluation protocol of cross-subject and cross-age, and details of experimental results, including single models, several combinations of ensemble, and models of previous works. 

### 4.1. Dataset

**Figure 14:**
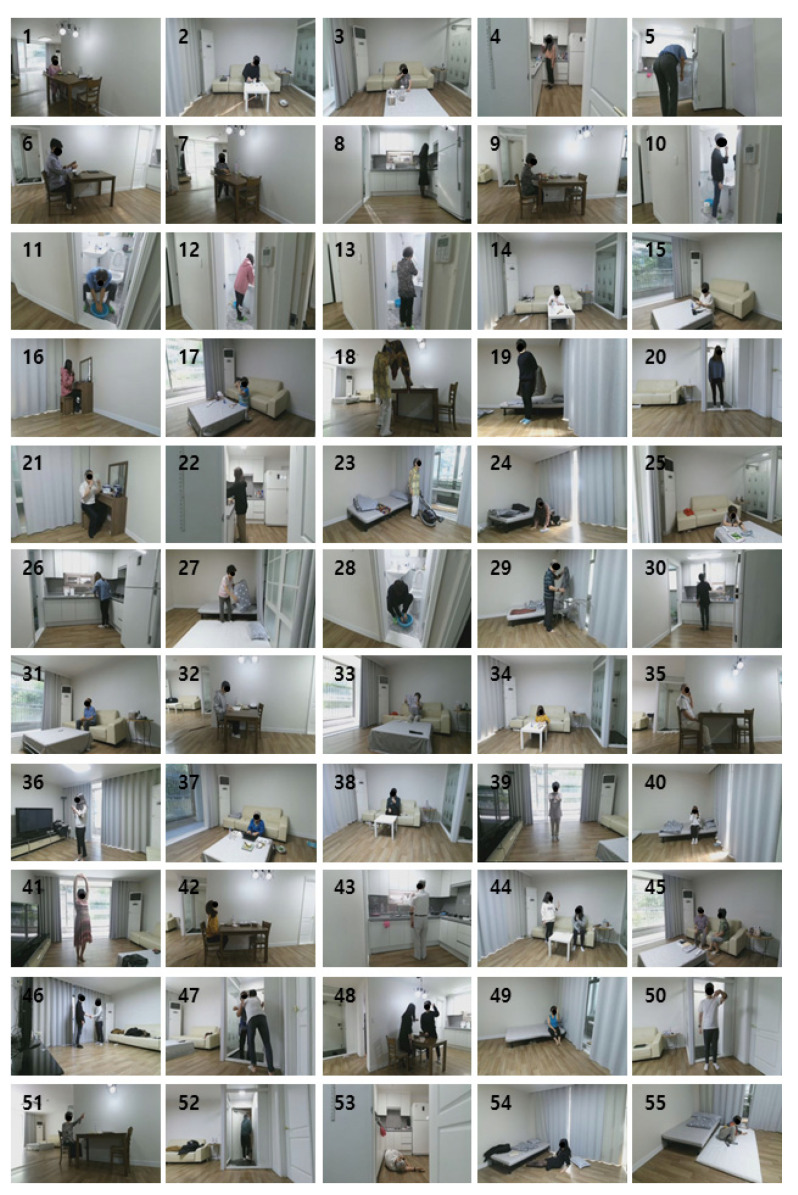
Examples of ETRI-Activity3D data.

The Electronics and Telecommunications Research Institute (ETRI)-Activity3D dataset was used to evaluate the behavior recognition performance of the proposed method. This is the second largest dataset with a total of 112,620 samples obtained from 50 elderly and 50 young individuals. The elderly individuals comprised 17 men and 33 women, averaging 77.1 years, with an age range from 64 to 88 years. The young individuals group consisted of 25 men and 25 women, averaging 23.6 years old, from 21 to 29 years old. Fifty-five actions were performed in daily life in the living room, kitchen, and bedroom in a residential apartment environment; they were acquired using Kinect v2. These actions were defined by observing actions that were performed frequently by elderly people in their daily life. Four Kinect sensors at heights of 70 and 120 cm were used to obtained data from eight directions, assuming a home service situation. The camera acquired photographs at a distance from 1.5 to 3.5 m from the subject. The format of the acquired data was 1920 × 1080 pixels for a color image and 512 × 424 pixels for a depth image, and the skeleton information included 25 joint positions in a 3D space. The frame rate of data was 20. The behavior types of ETRI-Activity 3D data are shown in Table 1, and the examples of ETRI-Activity3D data are as shown in Figure 14. For the diversity of data, actions that were performed 2–3 times by a person in different locations in the house (living room, bedroom, kitchen, etc.) or in different directions were acquired simultaneously by four or eight units from 100 individuals depending on the spatial conditions. There was an average of about 2050 datapoints for each action, and each person has an average of 20.5 datapoints [42]. Because the total size of the data was too large, the resolution was downsized by 1/5 to a resolution of 384 × 216.

### 4.2. Evaluation Methods

The organization of the data in training and testing followed the same manner as the previous experimental environments [42]. Thus, the dataset was divided into training and testing data for cross-subject and cross-age, respectively. After the hyperparameter was set, the training data was used to tune the parameters of the deep learning model. The testing data was used to check the performance only once after all tuning of the deep learning model was completed. This overall method has been tested under equivalent conditions based on a previously published base paper [42].

**Figure 15:**
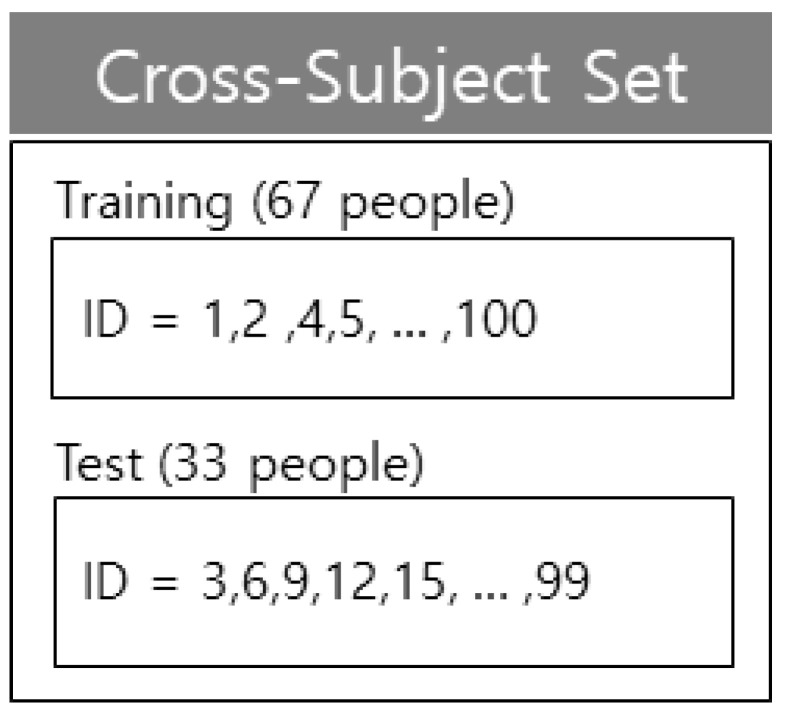
Composition of the CS set in ETRI-Activity3D.

**Figure 16:**
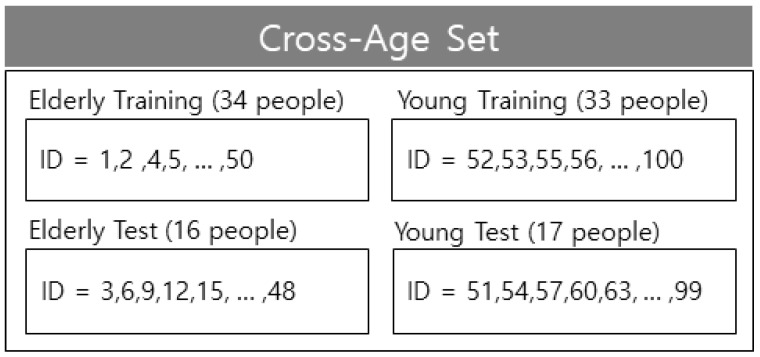
Composition of the CA set in ETRI-Activity3D.

Based on ETRI-Activity3D, numbers excluding the multiples of one to three were separated as training data; the multiples of one to three were separated as test data from 1 to 50 for 50 elderly individuals and from 51 to 100 for 50 young individuals in terms of cross-subject (CS). The training data consisted of 67 people with a mixture of young and elderly individuals; the test data consisted of 33 individuals with a mixture of young and the elderly. cross-age (CA) consisted of the elderly and young separated as elderly training, elderly test, young training, and young test. As in CS, in terms of CA, when 50 elderly people were from 1 to 50 and young people were from 51 to 100, the numbers excluding the multiples of one to three were separated as training data. The multiples of one to three were separated as test data, whereas the domains were separated at the boundary between the elderly and young people between 50 and 51 [42]. Further, numbers excluding the multiples of two to three were separated as training data and the multiples of two to three were separated as test data when 50 elderly people were from 1 to 50, and 50 young people were from 51 to 100, for CS cross-validation. The cross-validation was performed once due to the large amount of data. The composition of CS of ETRI-Activity3 is shown in Figure 15, and the composition of CA of ETRI-Activity3D is shown in Figure 16.

### 4.3. Experimental Results

The improvement of the accuracy compared to the conventional method was examined by classifying 55 behaviors of ETRI-Activity3D with the proposed method. The configuration of the device used in the experiment was as follows: Intel(R) Xeon(R) Gold 5120 2.2 GHz CPU (Central Processing Unit); NVIDIA Tesla V100-SXM2-32GB for GPU (graphics processing unit); 180 GB RAM (random access memory) capacity; and 64bit-based Window Server 2016 OS (operating system). The behavior recognition dataset is usually composed of RGB images, depth images, and skeletons. These can be recorded simultaneously because sensors that acquire these data are built into a single device. Information from various sensors can help improve accuracy because it provides more information in data analysis. Therefore, the characteristics of data can be optimally harmonized by designing recognizers that use the RGB video image and the skeleton sequence separately, and through ensemble of the scores of each recognizer at the end. First, a recognizer that uses RGB video was considered and then a recognizer that uses the skeleton sequence. The RGB video recognizer was designed using the 3D-CNN, and the skeleton sequence recognizer was designed using the PEI-2D-CNN method. 

**Figure 17:**
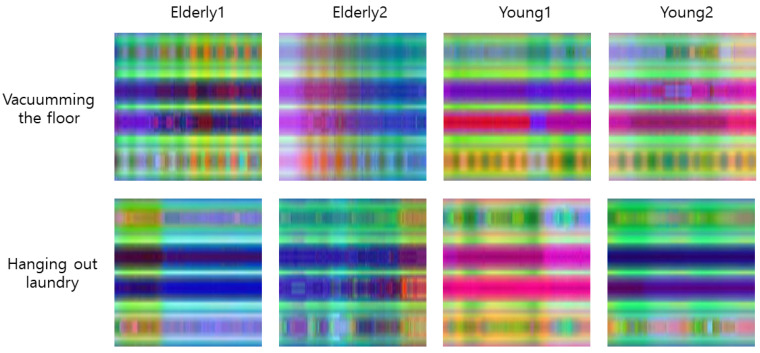
Example of PEI-T3 conversion from a skeleton.

**Figure 18:**
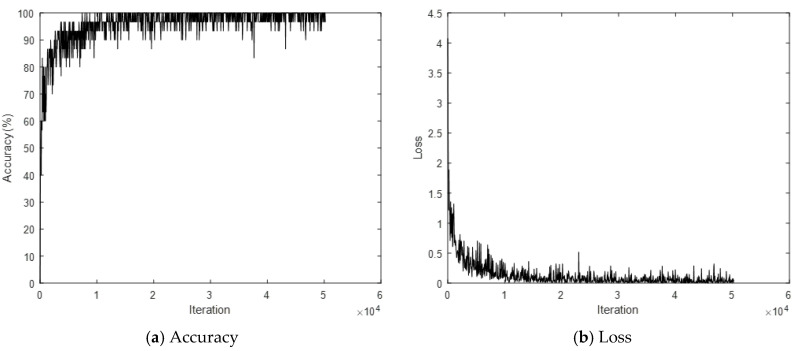
Graph depicting learning process of PEI-T3-2D-CNN.

The PEI-T1-2D-CNN method, which converts a 3D skeleton sequence into an image through the PEI method, and then classifies the image after inputting it into the 2D-CNN, is denoted as T1–T4 for the PEI types 1–4. The converted image is 224 × 224 × 3 RGB. The 2D-CNN uses ResNet101, which is a pre-trained model. The optimization method is Adam, the mini-batch size is 30, the initial learning rate is 0.0001, and the epoch is 20. An example of PEI-T3 conversion from a skeleton is shown in Figure 17; the skeleton-based behavior recognition accuracy (CS) is shown in Table 2. A graph of the learning process of PEI-T3-2D-CNN is shown in Figure 18.

Behavior recognition based on the ROI performs recognition by leaving only the ROI among the entire image region before analyzing the RGB video. The body ROI is a method of focusing on the object of interest by leaving only the person in the image. The hand–object ROI considers only the hand region to focus on the tools used by the human from the entire image. Two-dimensional joint coordinates were first obtained using OpenPose. A certain range around the coordinates was cropped and pasted into the same coordinates of a blank image of the same size without a background to leave only the human part. Only the human part was left, and the rest appears black once this process was completed for all joints. In the case of hand objects, it was performed only for the hand joints instead of all joints. Once this process was completed for all RGB videos, the ROI-based behavior recognition was performed. The prepared data were input into the 3D-CNN for recognition and training. 

**Figure 19:**
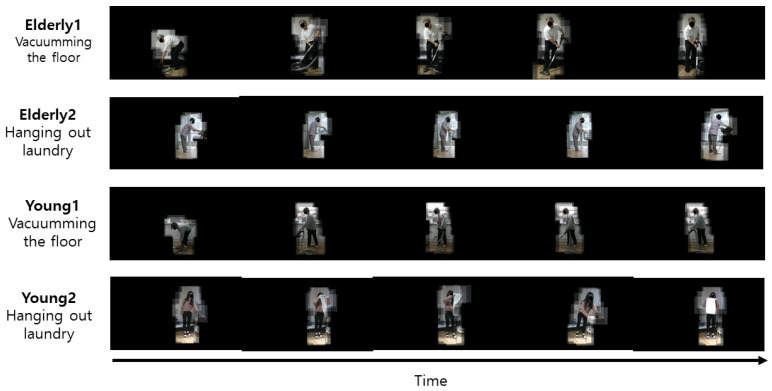
Example of body ROI video with only the body left and the background removed.

**Figure 20:**
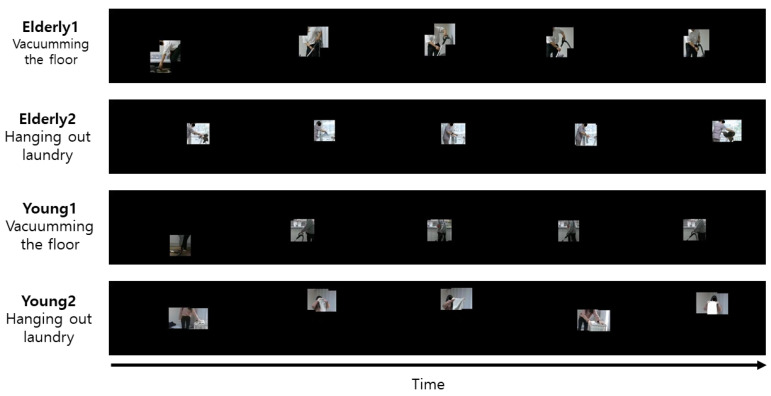
Example of hand–object ROI video with only the hand–object left and the background removed.

**Figure 21:**
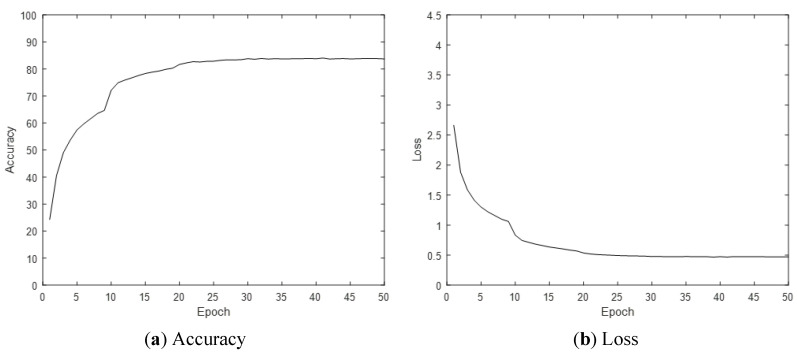
Graph of body ROI-based 3D-CNN learning process.

The 3D-CNN method is a CNN, where input data are 3D, and filters designed inside for feature extraction are 3D. The 3D convolution operation can learn better than the 2D-CNN on sequence data, because features are computed not only in the spatial domain, but also in the time domain. The 3D-CNN uses the pre-learning model of R3D-18 as a feature extractor and classifier of 3D data. The epoch is 50, the learning rate is 0.001, the optimization method is Adam, the weight decay is 0.00005, and the mini-batch size is 100. Accuracy is used as the performance evaluation metric of the behavior recognition model. An example of a body ROI video with only the body left and the background removed is shown in Figure 19, and an example of a hand–object ROI video with only the hand–object left and the background removed is shown in Figure 20. The accuracy (CS) of ROI-based behavior recognition is shown in Table 3 and a graph of the body ROI-based 3D-CNN learning process is shown in Figure 21.

Because the 3D-CNN was newly trained by focusing on the body ROI, which has the main information for behavior recognition in RBG video, different features were extracted, and different analysis was performed than when training with the entire image. Moreover, different features were extracted, and different analysis was performed than when training with the entire image because new learning is performed by focusing on the hand–object ROI, which has the main information for behavior recognition in the RGB video. Although the performance alone may be slightly degraded, because information is also removed from RGB in ROI, the performance expected if the information of these various results of the analysis is ensembled is far beyond the single performance of these methods. The ensemble result (CS) of the ROI-based model is shown in Table 4. ROIEnsAddNet1 and ROIEnsMulNet1 of ROI-based ensemble network are models obtained by adding and multiplying the results of the 3D-CNN of the RGB video input and the 3D-CNN of the body ROI input, respectively. ROIEnsAddNet2 and ROIEnsMulNet2 of ROI-based ensemble networks are models obtained by adding and multiplying the results of the 3D-CNN of the RGB video input, the 3D-CNN of the body ROI input, and the 3D-CNN of the hand–object ROI input, respectively. ROIEnsAddNet3 and ROIEnsMulNet3 of the ROI-based ensemble network are models obtained by adding and multiplying the results of the 3D-CNN of the RGB video input, the 3D-CNN of the body ROI input, the 3D-CNN of the hand–object ROI input, and the PEI-T3-2D-CNN. ROIEnsAddNet4 and ROIEnsMulNet4 of the ROI-based ensemble network are models obtained by adding and multiplying the results of the 3D-CNN of the RGB video input, the 3D-CNN of the body ROI input, the 3D-CNN of the hand–object ROI input, and the PEI-T1–T4-2D-CNN. The ROIEnsMulNet3 exhibits higher accuracy than the ensemble results of other combinations.

The results (CS) from comparing the conventional behavior recognition methods with the proposed behavior recognition method are shown in Table 5. Based on the previous experimental setup [42], we used the same training and testing dataset. Thus, these experimental results are compared in Table 4 and Table 5, respectively. The default value of open source was used without modification. Adam was used as the optimization method. The learning rate was randomly set from 1/3 to 3 times at every iteration until weight decay, and it was lowered by 1/3 from 0.001 to 0.000001 beyond weight decay. Various mini-batch sizes were trained by randomly setting it from 1 to 1/4 times based on the maximum GPU memory at each iteration. The performance of the proposed ROIEnsMulNet3 improved by a minimum of 4.27% and a maximum of 20.97% compared to other conventional methods. The ensemble result (CA) of the ROI-based model is shown in Table 6. In Table 6, the elderly training, young training, elderly test, and young test are also shown in Figure 16.

## 5. Conclusions

Behavior recognition was conducted using deep learning based on body and hand–object ROIs. Video-based behavior recognition is a technology that automatically detects the behavior of a target person through digital data processing. It can be applied to video-based automatic crime monitoring, automatic sports video analysis, and context awareness of a silver robot. In particular, the importance of research on behavior recognition as a core technology is increasing with the increase in the need for silver robots to solve the problem of elderly care due to the aging society. Behavior recognition data is mainly composed of images and skeletons, and better recognition performance can be expected by combining the analysis of data with different features. The important information in behavior recognition is the person performing the action. Therefore, feature analysis can be performed by focusing on the behavior itself rather than when training with the entire region by training the neural network after removing surrounding noise and placing the ROI on the person. Moreover, because humans use tools to perform actions, unlike animals, feature analysis can be performed focusing on tool information when a neural network is trained by placing an ROI on a hand–object interaction. Better performance can be expected by combining information from models that have been trained focusing on these regions of interest. The dataset used for the experiment was ETRI-Activity3D, which contains color images, images of skeletons, and depth images of 55 daily behaviors of 50 elderly and 50 young people. As a result of the experiment, the performance of the proposed ROI-based ensemble model improved by a minimum of 4.27% and a maximum of 20.97% compared to other behavior recognition methods. For future research, we will study effective information fusion approaches, comparing them with various methods of combining recognition results. Moreover, because it is necessary to consider the overfitting problem of learning including the validation data set, we will perform this experimental method in the future.

## Figures and Tables

**Table 1:** Behavior type of Electronics and Telecommunications Research Institute (ETRI)-Activity3D data.

1	eating food with a fork	29	hanging laundry
2	pouring water into a cup	30	looking around for something
3	taking medicine	31	using a remote control
4	drinking water	32	reading a book
5	putting (taking) food in (from) the fridge	33	reading a newspaper
6	trimming vegetables	34	writing
7	peeling fruit	35	talking on the phone
8	using a gas stove	36	playing with a mobile phone
9	cutting vegetable on the cutting board	37	using a computer
10	brushing teeth	38	smoking
11	washing hands	39	clapping
12	washing face	40	rubbing face with hands
13	wiping face with a towel	41	doing freehand exercise
14	putting on cosmetics	42	doing neck roll exercise
15	putting on lipstick	43	massaging a shoulder oneself
16	brushing hair	44	taking a bow
17	blow drying hair	45	talking to each other
18	putting on a jacket	46	handshaking
19	taking off a jacket	47	hugging each other
20	putting (taking) on (off) shoes	48	fighting each other
21	putting (taking) on (off) glasses	49	waving a hand
22	washing the dishes	50	flapping a hand up and down
23	vacuuming the floor	51	pointing with a finger
24	scrubbing the floor with a rag	52	opening the door and walking in
25	wiping off the dining table	53	falling on the floor
26	rubbing up furniture	54	sitting (standing) up
27	spreading (folding) bedding	55	lying down
28	washing a towel by hand		

**Table 2:** Skeleton-based behavior recognition accuracy (CS).

Method	Accuracy (%)
Skeleton (PEI-T1-2D-CNN)	84.95
Skeleton (PEI-T2-2D-CNN)	85.88
Skeleton (PEI-T3-2D-CNN)	86.09
Skeleton (PEI-T4-2D-CNN)	85.20

**Table 3:** ROI-based behavior recognition accuracy (CS).

Method	Accuracy (%)
RGB (3D-CNN)	79.20
Body ROI RGB (3D-CNN)	76.85
Hand–object ROI RGB (3D-CNN)	73.11

**Table 4:** Ensemble results (CS) of the ROI-based model.

Method	Accuracy (%)
ROIEnsAddNet1	84.68
ROIEnsAddNet2	86.83
ROIEnsAddNet3	92.79
ROIEnsAddNet4	94.18
ROIEnsMulNet1	85.85
ROIEnsMulNet2	87.98
ROIEnsMulNet3	94.87
ROIEnsMulNet4	94.69

**Table 5:** Performance comparison with conventional behavior recognition methods (CS).

Method	Accuracy (%)
IndRNN [22]	73.90
Beyond Joints [23]	79.10
SK-CNN [24]	83.60
ST-GCN [25]	86.80
Motif ST-GCN [43]	89.90
Ensem-NN [44]	83.00
MANs [45]	82.40
HCN [46]	88.00
FSA-CNN [42]	90.60
ROIEnsMulNet3	94.87

**Table 6:** Ensemble results (CA) of ROI-based model.

Method		Accuracy (%)	
		Elderly Test	Young Test
FSA-CNN [42]	Elderly Training	87.70	69.00
	Young Training	74.90	85.00
ROIEnsAddNet3	Elderly Training	92.53	70.35
	Young Training	73.57	89.87
ROIEnsMulNet3	Elderly Training	94.57	75.04
	Young Training	79.51	92.54

## Data Availability

Not applicable.
